# *Osmundastrum cinnamomeum* population differences in three habitat types in South Korea

**DOI:** 10.1016/j.heliyon.2024.e33183

**Published:** 2024-06-16

**Authors:** Ho Yeong Yu, Jae Geun Kim

**Affiliations:** a Department of Science Education, Graduate School, Seoul National University, Seoul, 08826, Republic of Korea; b Department of Biology Education, Seoul National University, Seoul, 08826, Republic of Korea; c Center for Education Research, Seoul National University, Seoul, 08826, Republic of Korea

## Abstract

Most ferns occur in moist and shaded environments; their relatively narrow band of survival conditions increase their extinction risk under Anthropocene-linked climate change. *Osmundastrum cinnamomeum* (L.) C. Presl is a perennial fern that has a natural distribution primarily encompassing the East Asian mountains. In this study, we characterized the vegetation and environmental factors in low- and high-elevation mountain ranges and montane wetlands to elucidate the optimal habitat for *O. cinnamomeum*. We found that populations of *O. cinnamomeum* developed better in high-elevation mountains than in low-elevation mountains. However, the low light intensity in high mountain forests reduces opportunities for reproduction. The montane wetlands recorded the highest biomass among the three habitat types, and the investment in reproduction was also significantly higher. The montane wetlands also showed higher light intensity, substrate fertility, and water content. Overall, the montane wetlands were found to be the optimal habitat for *O. cinnamomeum*. At lower elevations, lower precipitation, higher temperatures, human disturbance, and low substrate water could be factors limiting the expansion of this fern's distribution. Our study points to the importance of conserving montane wetlands to prevent the extinction of ferns.

## Highlights

• Montane wetland is the optimal habitat of the *Osmundastrum cinnamomeum*.
• Anthropogenic disturbances and high temperatures threaten Korean montane wetlands.
• *Osmundastrum cinnamomeum* could become extinct due to habitat loss.

## 1 Introduction

Studies into how population structure and size are related to different environmental and geographic factors can provide valuable information about the ecological needs and life history strategies of species [[1], [2], [3]]. The International Panel on Climate Change (IPCC) predicted that temperatures will increase by 1–3.7 °C by 2100 [4]. This change is expected to disrupt the habitats of numerous plants at local scales, potentially resulting in their extinction [5].

Most ferns are known to prefer moist and shaded environments [6]; this relatively narrow band of appropriate conditions for survival, makes ferns highly vulnerable to habitat changes [7]. Specifically, the physiology of ferns has resulted in their stomatal regulatory processes being less efficient and slower than those of angiosperms [8]. Additionally, since ferns reproduce through spores rather than seeds, their reproduction and growth are greatly determined by environmental variables, i.e., temperature and precipitation, more so than angiosperms [6,9].

Mountains are known to be centers of fern diversity [6]. Mountainous regions exhibit a lot of variation in terms of elevation, topography, and climate, providing an ideal environment to host a wide array of fern species [6]. The distribution and growth of pteridophytes in mountainous areas vary based on elevation [10]. Specifically, climatic and edaphic factors that change with elevation are known to influence the population structure and size of pteridophytes [11]. For instance, studies in Costa Rica and Panama found that water scarcity at low elevation and low temperatures at high elevation were the main determinants of fern abundance [12].

In mountains, local-scale environmental characteristics, such as edaphic factors, can vary considerably depending on climate and geographical features and are significant to ferns [10]. In central Italy, it has been reported that high moisture content determines the habitat suitability of numerous fern species [3], with ferns being used as indicators of high moisture at microhabitat-scales [13].

Several studies suggest that the survival strategies of ferns are shaped by complex interactions of several environmental variables. For example, the density of *Osmunda regalis* in spring marshes was found to be positively correlated with elevation but negatively affected by shrub cover [1]. However, there is still no comprehensive understanding about the survival strategies of ferns in high-elevation environments [6,10,14], and further research is needed on the population structure of these ferns across different habitat types in high-elevation contexts [15].

In South Korea, where 64 % of the land is mountainous, fern biodiversity is threatened by climate change and anthropogenic disturbances such as logging; these trends have reduced the potential habitat area of ferns [16]. *Osmundastrum cinnamomeum* (L.) C. Presl is a representative fern found in the mountains of Korea [17,18]. This fern is threatened by habitat loss and anthropogenic extraction [19]. *O. cinnamomeum* is distributed across a wide range of elevation, from relatively low to high elevations, making it an excellent model for investigating ecological characteristics of ferns that change with elevation. In this study, we (1) elucidate the environmental conditions that characterize the habitat conditions of *O. cinnamomeum* populations in Korean mountains and determine how they shape the development and distribution patterns of the species. Additionally, we (2) also determine the optimal habitat conditions for these fern populations. Through such species-environment studies, we can better understand competition and adaptation strategies in mountain forests and montane wetlands and provide insights that can be applied toward the conservation of *O. cinnamomeum*.

## 2 Materials and methods

### 2.1 Model species

*Osmundastrum cinnamomeum* (L.) C. Presl, a fern native to East Asia and America, is known as a ‘living fossil’ because it has been in evolutionary stasis for approximately 200 million years [18]. *O. cinnamomeum* is the only living species and is classified as a single lineage in the genus *Osmundastrum*, based on molecular and morphological fossil evidence [20]. *O. cinnamomeum* is a dimorphic fern with both sterile and fertile fronds [18,21]. It prefers moist habitats and is an indicator species in wet environments [22]. *O. cinnamomeum* is a dominant member of plant communities in fens and bogs [[23], [24], [25]]. *O. cinnamomeum* is used as a medicinal material to treat the hemostatic effect of intestinal bleeding in oriental medicine [26], and is known as a natural antidiabetic material with α-glucosidase inhibitory activity [19]. In Asia, it is used for dietary and medicinal purposes [27,28], and is traded at a high price due to its scarcity in relation to its demand [19]. *O. cinnamomeum* faces extinction risk in low-latitude Asian regions such as Taiwan [29]. For the conservation of *O. cinnamomeum*, extensive research has been conducted on its germination ability [[29], [30], [31]].

### 2.2 Study sites

**Fig. 1:**
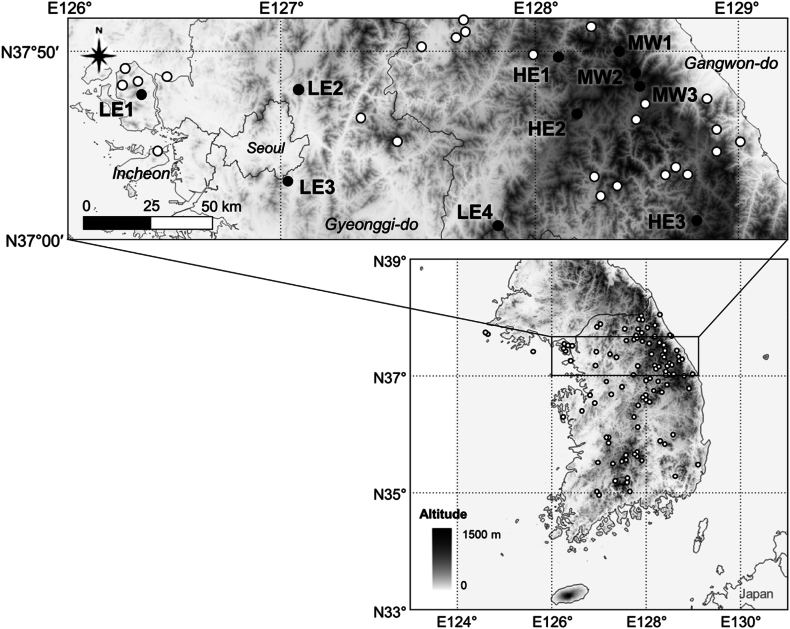
Study sites (filled circles) and distribution of *Osmundastrum cinnamomeum* habitat in the Republic of Korea based on a Korea Biogeographic Information System [32] (empty circles). Shaded areas represent the elevation (in the grey scale, darker areas are higher). LE1, Incheon; LE2, Uijeongbu; LE3, Seongnam; LE4, Jecheon; HE1, Hongcheon; HE2, Inje; HE3, Jeongseon; MW1, Hongchen (Jogaedong-neup wetland); MW2, Pyeongchang (Sowhangbyungsan-neup wetland); and MW3, Pyeongchang (Jilmoe-neup wetland). LE are low elevation mountains; HE are high elevation mountains; MW are montane wetlands protected by Ramsar Convention.

According to the distribution map of *O. cinnamomeum* based on the Korea Biogeographic Information System [32], *O. cinnamomeum* is distributed in the major mountain ranges in Korea (Fig. 1). We selected 10 sites in three habitat types based on the life history of *O. cinnamomeum*, elevation, and landscape characteristics of the research site (Table 1): low-elevation mountains—Incheon (LE1), Uijeongbu (LE2), Seongnam (LE3), Jecheon (LE4); high-elevation mountains—Hongcheon (HE1), Inje (HE2), Jeongseon (HE3); and montane wetlands protected by the Ramsar Convention—Hongchen Jogaedong-neup (MW1), Pyeongchang Sowhangbyungsan-neup (MW2), and Pyeongchang Jilmoe-neup (MW3).

**Table 1:** Geographical characteristics for the study sites of *Osmundastrum cinnamomeum*. Soil and parent rock layer characteristics are based on Korean Soil Information System [33,34]. The annual mean temperatures for the study sites are detailed in Table S2.

Site (number of plots)	Elevation (m)	Symbol	Latitude (N)	Longitude (E)	Soil texture	Soil depth (cm)	Parent rock layer	Geography
Incheon (n = 8)	47–60	LE 1	37°40′N	126°29′E	Loams	50–100	Metamorphic rock	Slope, near hiking trail
Uijeongbu (n = 7)	187–230	LE 2	37°42′N	127°04′E	Sandy loams	0–20	Acidic rock	Valley, near hiking trail
Seongnam (n = 5)	409–418	LE 3	37°24′N	127°03′E	Sandy loams	50–100	Metamorphic rock	Slope, deep in woods
Jecheon (n = 6)	372–388	LE 4	37°12′N	127°58′E	Sandy loams	50–100	Metamorphic rock	Valley, near hiking trail
Hongcheon (n = 5)	971–988	HE 1	37°50′N	128°19′E	Sandy loams	0–20	Acidic rock	Slope, deep in woods
Inje (n = 2)	675–695	HE 2	37°43′N	128°21′E	Sandy loams	0–20	Metamorphic rock	Slope, near graves
Jeongseon (n = 6)	1288–1311	HE 3	37°09′N	128°54′E	Silty loams	0–20	Sedimentary rock	Slope, near hiking trail
Hongcheon (Jogaedong-neup) (n = 5)	772–792	MW 1	37°50′N	128°33′E	Sandy loams	0–20	Acidic rock	Slope, designated as Ramsar wetlands
Pyeongchang (Sowhangbyungsan-neup) (n = 6)	1168–1180	MW 2	37°46′N	128°40′E	Loams	50–100	Acidic rock	Valley, designated as Ramsar wetlands
Pyeongchang (Jilmoe-neup) (n = 6)	1049–1058	MW 3	37°46′N	128°42′E	Loams	50–100	Acidic rock	Slope, designated as Ramsar wetlands

LE sites are at longitudes of 126∼127 °E and an elevation of 47∼418 m in elevation in Korea (Fig. 1). The HE and MW sites are at 128 °E longitude and an elevation of 675–1311 m and 772–1180 m, respectively. MW sites are protected as Ramsar wetland in Odaesan National Park and are in wet meadows or at the edge of forest land. LE and HE sites are deep in the forest or at the edge of a forest where there is a small stream nearby or the land is temporarily wetted by surface water. Most of the soil in the habitat of *O. cinnamomeum* habitat was sandy loam or loam. In addition, the habitat also exhibited heterogeneity in terms of soil depth (Table 1). At LE sites, the parent rock layer was mostly metamorphic rock, MW sites were acidic rock, and HE hosted a wide range of rock types [33,34].

### 2.3 Field data collection

Field surveys were conducted twice, specifically, in May (spore season) and August (peak of the growing season) of 2021. To collect data on the population development and community structure of *O. cinnamomeum*, 2 × 2 m plots were established at each research site. The population was located at the edge of the forest, and the size of the plot was determined considering the size of the major species. The plots were randomly installed where population of *O. cinnamomeum* grew, and the number of plots varied among the sites depending on the population size (Table 1). The numbers of plots at the LE, HE, and MW sites were 26, 13, and 17, respectively. The density was determined by counting the number of ramets, and the coverage and mean height of the plants were measured. The relative density, relative coverage, and frequency were calculated [35]. According to Choung et al. [36], other vascular species present in the plots were classified into five categories based on the frequency of each species in its habitat as follows: obligate upland plants (OBU), facultative upland plants (FACU), facultative plants (FAC), facultative wetland plants (FACW), and obligate wetland plants (OBW). The number of fertile fronds per plot was determined in May, which is the spore season for *O. cinnamomeum* in Korea. The species richness represented the number of species present at the study sites. Species evenness and species diversity were calculated using the Pielou index [37] and Shannon–Wiener index [38], respectively. The importance values of each species were determined by calculating the average level of relative coverage and relative density. We identified the studied plants using the WFO Plant List (https://www. wfoplantlist.org/) and Coloured Flora of Korea [39]. Sampling was minimized in the study area, as it encompasses protected zones. Ho Yeong Yu took pictures of all the plants for certification according to the national regulation. Samples of *O. cinnamomeum* were collected as voucher specimens to be preserved in the Herbarium of Department of Biology Education, Seoul National University, Seoul, Korea (No. 23YHYOCO1, 23YHYOCO2).

The environmental properties of each study site were investigated. The light intensity was measured at the top of individual *O. cinnamomeum* plants at three spots in each plot and open area using a light intensity meter (LI-250 A Light Meter; LI-COR Biosciences, Lincoln, NE, USA), and the relative light intensity (RLI) was calculated. Owing to overcast weather, the August RLI of the MW2 and MW3 wetlands protected by the Ramsar Convention could not be measured. Therefore, some RLI values were calculated from the canopy cover according to the following equation:

$$y=100e^{-0.02x}$$

where *x* is the canopy coverage (%), *y* is the RLI (%), and the constants were estimated from the field survey data in May (*R*^2^ = 0.8325). The elevation and slope angle were measured within each plot. Substrate samples were collected from each plot at a depth of 0–10 cm, where rhizosphere of *O. cinnamomeum* occurs. The samples were sealed in plastic bags for subsequent physicochemical analyses in the laboratory. The annual mean temperature and the monthly mean precipitation from 2012 to 2021 of the three habitat types were determined to characterize and compare the climatic conditions of the three habitat types [40]. Climate data were collected from the automated weather stations closest to the study sites (Table S3).

### 2.4 Analysis of substrate properties

After the substrate samples arrived at the laboratory, they were passed through a 2-mm pore size sieve (standard sieve #10). The water content of these samples was measured by drying them at 105 °C for more than 48 h in a dry oven [35], whereas their organic content was determined using the loss on ignition method [41]. The pH and electrical conductivity were measured using a pH meter (Starter 300C; OHAUS, Parsippany, NJ, USA) and a conductivity meter (Portable AP63 Meter; Accumet, Westford, MA, USA), respectively, after mixing the dried substrate and adding deionized water at a mass ratio of 1:5 and filtering the solution through filter paper. NO_3_–N and NH_4_–N were extracted with 2 M potassium chloride solution, and the contents were determined according to the hydrazine [42] and indophenol [43] methods, respectively. The PO_4_–P content was quantified using the ascorbic acid reduction method [44] after extraction with a Bray No.1 solution [45]. The cations (K^+^, Na^+^, Ca^2+^, and Mg^2+^) were extracted with 1 N ammonium acetate (CH_3_COONH_4_) solution, and their concentrations were measured using an atomic absorption spectrometer (AA240FS: Varian, Palo Alto, Ca, USA).

### 2.5 Growth measurement

On August of 2021, the growth characteristics of *O. cinnamomeum* were measured at the respective study sites. The number of fronds was counted for each ramet in the plot. Fronds with an average width were selected and collected from each ramet. The collected fronds were then transported to the laboratory. In the laboratory, petiole diameter and frond length were measured. The frond area was measured by LI-3000C and LI-3050C transparent belt conveyers (LI-COR Biosciences). The frond samples were dried at 60 °C for 3 days, and weighed to determine dry weight.

### 2.6 Statistical analysis

Detrended canonical correspondence analysis (DCCA), using CANOCO4.5 software [46], was used for vegetation ordination and to determine the relationship between the distribution of *O. cinnamomeum* populations and environmental variables. Species matrices were developed including 84 (in May) and 89 (in August) for species that had a higher frequency than five in herb, shrub, or tree layers [47], and 56 plots were built using IV for each species, which were used in DCCA. Environmental matrices were built for 16 factors and 56 plots using the measured values of topography and substrate properties in May and August. Repeated measures analysis of variance (RMANOVA) was used to analyze the changes in environmental and vegetation characteristics according to habitat type (LE, HE, and MW) and season (May and August). All variables were subjected to a one-way analysis of variance (ANOVA) to analyze the differences between habitat types for each season. Statistical differences between the groups were determined using Scheffe's post-hoc test. The Kolmogorov-Smirnov test was used to determine if the data distributions departed significantly from normality. RMANOVA, ANOVA analyses and K–S test were conducted using SPSS version 28 (IBM Corp., Armonk, NY, USA) at a 95 % significance level.

## 3 Results

### 3.1 Habitat types according to environmental variables and species composition

We observed eight, nine, and 74 species in the tree, shrub, and herb layers, respectively, as the major co-occurring species in the habitats of *O. cinnamomeum* (Table S1). Among the plant species observed, obligate upland plant, facultative upland plant, facultative plant, facultative wetland plant, and obligate wetland plants accounted for 57.8 %, 3.6 %, 14.5 %, 15.7 %, and 8.5 %, respectively. Based on habitat type, the major co-occurring species in the Low Elevation mountains (LE) sites were *Quercus mongolica* (frequency 42 %) and *Stephanandra incisa* (frequency 34 %) in May and *Oplismenus undulatifolius* (frequency 50 %) and *Athyrium yokoscense* (frequency 42 %) in August; the major co-occurring species in High Elevation mountains (HE) were *Carex siderosticta* (frequency 76 %) and *Angelica dahurica* (frequency 53 %) in May and *Carex siderosticta* (frequency 76 %) and *Aconitum pseudolaeve* (frequency 53 %) in August; the major co-occurring species in Montane Wetlands (MW) were *Spiraea fritschiana* (frequency 64 %), *Thelypteris palustris* (frequency 58 %), and *Salix gracilistyla* (frequency 41 %) in May and *Thelypteris palustris* (frequency 82 %), *Spiraea fritschiana* (64 %), *Gentiana triflora* (52 %) in August. Overall, most co-occurring species were upland plants in LE and HE sites, and wetland plants in MW sites.

**Fig. 2:**
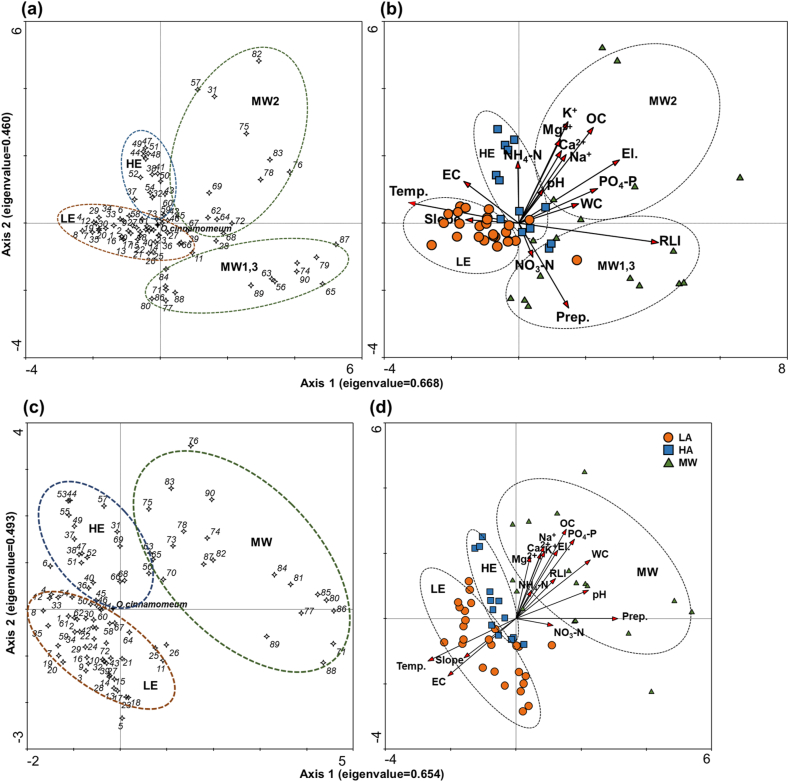
Detrended canonical correspondence analysis (DCCA) ordination of the habitat of *Osmundastrum cinnamomeum* based on the species matrix of 84 species and 56 plots in May (a), and on the environment matrix of 16 factors and 56 plots in May (b), species matrix of 89 species and 56 plots in August (c), and on the environment matrix of 16 factors and 56 plots in August (d), Korea (species name for each number described in Table S1). Different cycles in the DCCA figure represent different sites. Specifically, LE are low elevation mountains, HE are high elevation mountains, and MW are montane wetlands protected by Ramsar Convention. RLI, relative light intensity; WC, water content; OC, organic content; EC, electrical conductivity; El, elevation.

In May, a DCCA was conducted based on the species matrix of 84 species and the environmental matrix of 16 environmental variables in 56 plots (Fig. 2a and b). The eigenvalues of the first two DCCAs were 0.668 and 0.460, respectively, explaining 26.5 % of the variance. The correlation score for the species–environment relationship was 0.964 on the first axis and 0.926 on the second axis. The first DCCA axis was strongly related to the RLI, elevation, and temperature (Table 2). The relative light intensity and elevation of habitats increased from left to right, and the annual mean temperature decreased from left to right. According to the first axis, the habitat types of *O. cinnamomeum* are classified as LE, HE, and ‘MW1, MW2, MW3’ (Fig. 2a and b). The second DCCA axis represents a gradient in organic carbon and K^+^ content, increasing from the bottom to the top of the y-axis. Correspondingly, the habitat types and plant communities of *O. cinnamomeum* were divided into ‘MW1 and MW3’, LE, HE, and MW2 (Fig. 2a and b).

**Table 2:** Correlation coefficient matrix between environmental variables and axes of detrended canonical correspondence analysis plots in Fig. 2.

Variables	Correlation coefficient			
	May		August	
	Axis 1	Axis 2	Axis 1	Axis 2
RLI	0.8531	−0.0930	0.3423	0.2875
El.	0.6158	0.3027	0.3616	0.4894
Slope	−0.3254	0.0172	−0.4490	−0.2805
Temp.	−0.6759	0.0986	−0.7607	−0.3037
Prep.	0.3045	−0.4065	0.8823	−0.0006
WC	0.3656	0.0934	0.6443	0.4199
OC	0.4555	0.4600	0.4372	0.6409
pH	0.1521	0.1656	0.6287	0.2021
EC	−0.3368	0.2012	−0.5898	−0.4121
NH_4_–N	−0.0052	0.2968	0.1351	0.2017
NO_3_–N	0.0857	−0.1630	0.3222	−0.0487
PO_4_–P	0.4804	0.1668	0.5073	0.5693
Ca^2+^	0.2603	0.3477	0.2161	0.4682
K^+^	0.3012	0.4870	0.2505	0.4827
Na^+^	0.2894	0.3301	0.2432	0.5183
Mg^2+^	0.2524	0.4025	0.1231	0.4473

In August, the eigenvalues of the first two axes of DCCA based on the species matrix of 89 species and the environmental matrices of 16 environmental variables at 56 plots were 0.654 and 0.493, respectively (Fig. 2c and d). The correlation scores for the species–environment relationship were 0.951 and 0.927 in May and August, respectively, with the two axes explaining 26 % of the variance. The first DCCA axis is strongly explained by the precipitation, temperature, and substrate water content. On the x-axis, the monthly mean precipitation and the substrate water content increased from left to right, whereas the annual mean temperature decreased from left to right (Table 2). The second DCCA axis represents a gradient in organic carbon content and PO_4_–P content, increasing from bottom to top on the y-axis. According to the first two DCCA axes in August, the habitat types of *O. cinnamomeum* were divided into LE, HE, and MW from the lower left to the upper right (Fig. 2c and d).

### 3.2 Environmental properties of the habitat types

Statistically significant differences were observed between habitat types for most environmental factors but not seasonally (Table 3). As a result of the RMANOVA, RLI and OC showed significant differences by type (*p* < 0.001; Table 3) and showed the highest value in MW. WC and pH showed a type × season interaction (*p* < 0.001; *p* < 0.05; Table 3). WC was highest in MW in May and lowest in LE in August. The pH was significantly lower in LE in both May and August (*p* < 0.001; Table S2). The EC and PO_4_–P and NH_4_–N contents showed a type × season interaction (*p* < 0.05, *p* < 0.001, *p* < 0.05, respectively; Table 3). The EC had the highest value in HE in May and the highest value in LE in August. The PO_4_–P content showed the lowest value in LE in May and the highest value in MW in August. The NH_4_–N content was not significantly different in May, but the MW was significantly higher than the HE in August (*p* < 0.05; Table S2). The NO_3_–N content was significantly different between types (*p* < 0.01; Table 3), and was higher in HE and MW than in LE. The Ca^2+^ and K^+^ contents were significantly different in both type and season (Table 3): the Ca^2+^ content showed the lowest values in both May and August in the LE sites and increased in August compared to May. The K^+^ content decreased in August compared with May, with the lowest value in the LE sites in May and the highest value in the MW sites in August.

**Table 3:** Significance of differences in vegetative characteristics and environmental characteristics of *Osmundastrum cinnamomeum* habitat among three types of LE, HE, and MW in May and August. F statistics and *p*-values based on repeated measures analysis of variance are shown to highlight the effects of habitat types and season on vegetation characteristics and environmental characteristics. Statistically significant differences (*p* < 0.05) are presented in boldface.

Classification	Variable	Source	df	F	*p*
Environmental Characteristics	Relative light intensity (%)	Type	2	**20.220**	**0.000**
		Season	1	1.617	0.209
		Type*Season	2	0.654	0.524
	Water content (wt%)	Type	2	**44.985**	**0.000**
		Season	1	**8.878**	**0.004**
		Type*Season	2	**12.739**	**0.000**
	Organic content (%)	Type	2	**15.566**	**0.000**
		Season	1	0.230	0.633
		Type*Season	2	0.557	0.576
	pH	Type	2	**28.991**	**0.000**
		Season	1	0.741	0.393
		Type*Season	2	**3.558**	**0.035**
	Electric conductivity (μS cm^−1^)	Type	2	**23.813**	**0.000**
		Season	1	**25.649**	**0.000**
		Type*Season	2	**4.315**	**0.018**
	NH_4_–N (mg kg^−1^)	Type	2	1.205	0.308
		Season	1	**14.499**	**0.000**
		Type*Season	2	**3.177**	**0.049**
	NO_3_–N (mg kg^−1^)	Type	2	**6.718**	**0.003**
		Season	1	3.523	0.066
		Type*Season	2	0.182	0.834
	PO_4_–P (mg kg^−1^)	Type	2	**19.214**	**0.000**
		Season	1	0.086	0.771
		Type*Season	2	**12.150**	**0.000**
	Ca^2+^ (mg kg^−1^)	Type	2	**14.493**	**0.000**
		Season	1	**4.685**	**0.035**
		Type*Season	2	2.945	0.061
	K^+^ (mg kg^−1^)	Type	2	**12.061**	**0.000**
		Season	1	**15.750**	**0.000**
		Type*Season	2	2.948	0.061
	Na^+^ (mg kg^−1^)	Type	2	**12.669**	**0.000**
		Season	1	1.322	0.255
		Type*Season	2	**3.926**	**0.026**
	Mg^2+^ (mg kg^−1^)	Type	2	**11.682**	**0.000**
		Season	1	1.679	0.201
		Type*Season	2	1.364	0.264
Vegetation characteristics	Density (ramet/4 m^2^)	Type	2	**32.369**	**0.000**
		Season	1	2.162	0.147
		Type*Season	2	1.451	0.243
	Coverage (%)	Type	2	**4.623**	**0.014**
		Season	1	**31.470**	**0.000**
		Type*Season	2	2.889	0.064
	Height (cm)	Type	2	**22.886**	**0.000**
		Season	1	**10.399**	**0.002**
		Type*Season	2	0.046	0.955
	Shannon–Wiener Index	Type	2	**9.853**	**0.000**
		Season	1	**64.330**	**0.000**
		Type*Season	2	0.469	0.628
	Richness Index	Type	2	**16.756**	**0.000**
		Season	1	**31.977**	**0.000**
		Type*Season	2	1.371	0.263
	Pielou Index	Type	2	1.837	0.169
		Season	1	**19.609**	**0.000**
		Type*Season	2	0.380	0.686
	Importance value (%)	Type	2	2.677	0.078
		Season	1	**6.803**	**0.012**
		Type*Season	2	0.813	0.449

One-way ANOVA revealed a significant difference in annual mean temperature, with the highest in LE sites and lowest in MW sites (*p* < 0.001). The monthly mean precipitation was also significantly different and was the highest in the MW sites (*p* < 0.001). Elevation was significantly lower in LE than in the MW and HE groups (*p* < 0.001). The slope angles were significantly higher in the LE sites than in the HE and MW sites (*p* < 0.001; Table S2).

### 3.3 Comparison of community structure among habitat types

**Fig. 3:**
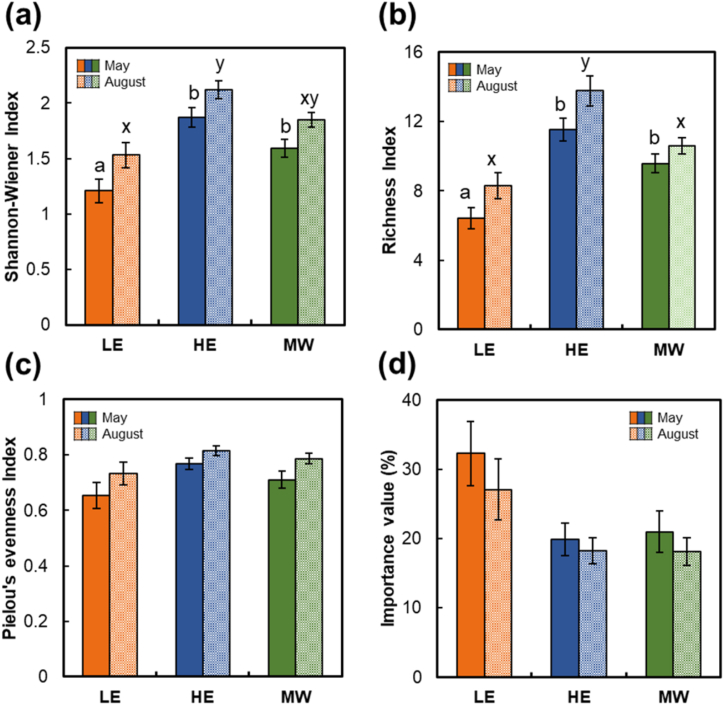
Community structure in the three habitat types in May and August (*n* = 26 for LE; *n* = 13 for HE; *n* = 17 for MW). (a) Shannon–Wiener index; (b) richness index; (c) Pielou index; (d) importance value of *Osmundastrum cinnamomeum*. Lowercase letters (a–c in May and x–z in August) on the graph represent statistically significant differences between types based on Scheffe's post hoc test (*p* < 0.05), and bars indicate standard error.

The community structure varied significantly between seasons (Table 3; Fig. 3). *O. cinnamomeum* was less important to the plant communities in August, when plant diversity indices and evenness were higher (Fig. 3a) than in May (Fig. 3d). According to our analysis, the Shannon-Wiener diversity index and the richness index were significantly higher in August than in May (*p* < 0.001), with this trend being especially pronounced in high elevation (HE) sites (Fig. 3b). This suggests that the richness of plant community changes with the seasons, particularly increasing during the warmer months.

Additionally, despite there being no significant difference in the Pielou evenness index among habitat types (Table 3; Fig. 3c), this index was significantly higher in August than in May (*p* < 0.001), indicating that the even distribution of species within these communities can vary seasonally. In the case of *O. cinnamomeum*, its importance decreased in August, which may indicate that it becomes relatively less important in competition with other plant species during periods of increased diversity.

**Fig. 4:**
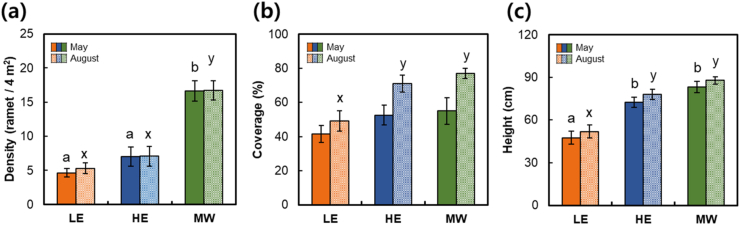
Population development status of *Osmundastrum cinnamomeum* in three types of habitat (*n* = 26 for LE, *n* = 13 for HE, and *n* = 17 for MW). (a) Density, (b) coverage, and (c) height of *O. cinnamomeum*. Lowercase letters (a–c in May and x–z in August) on the graph represent statistically significant differences between types based on Scheffe's post hoc test (*p* < 0.05), and bars indicate standard error.

The different habitats of *O. cinnamomeum* differed significantly in terms of habitat type (*p* < 0.001). Specifically, density of MW sites was three times greater than that in LE sites and more than two times greater than that in HE sites (Fig. 4a). In May, the habitats did not differ significantly in terms of the coverage of *O. cinnamomeum*; however, the coverage of HE and MW sites was significantly greater than that of LE sites in August (*p* < 0.001; Fig. 4b). The coverage and height of *O. cinnamomeum* were significantly higher in August (*p* < 0.001) than in May (*p* < 0.01). The height of *O. cinnamomeum* was significantly greater in the MW and HE sites than in the LE sites in both May and August (*p* < 0.001; Fig. 4c).

**Fig. 5:**
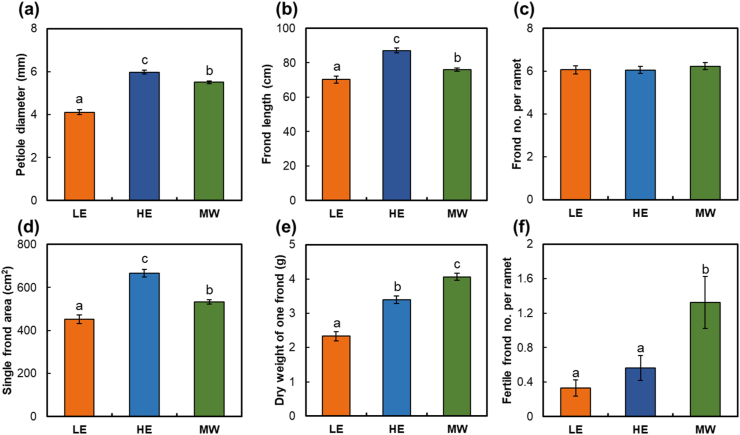
Growth and reproductive characteristics of *Osmundastrum cinnamomeum* for each habitat types (*n* = 138 for LE, *n* = 92 for HE, and *n* = 278 for MW). (a) Petiole diameter, (b) frond length, (c) number of fronds per ramet, (d) single frond area, (e) dry weight of one frond, (f) number of fertile fronds per ramet. Lowercase letters on the graph represent statistically significant differences between types based on Scheffe's post hoc test (*p* < 0.05), and bars indicate standard error.

One-way ANOVA results on the growth and reproduction characteristics of *O. cinnamomeum* showed significant differences by habitat type (*p* < 0.001), except for the number of fronds per ramet. The petiole diameter, frond length, and single frond area were highest and lowest in HE and LE sites, respectively (Fig. 5a, b, d). However, the number of fronds per ramet was almost 6, with no significant difference by habitat type (Fig. 5c). The dry weight of one frond of an MW plant was significantly higher than that of a HE or LE plant (Fig. 5e). This means that the frond thickness of MW plants is greater than that of HE plants. In addition, the dry weight per plot calculated considering the density of MW plants was more than four times higher than that of LE plants and more than three times higher than that of HE plants (*p* < 0.001; Fig. S1). The number of fertile fronds per ramet of *O. cinnamomeum* surveyed for reproductive characteristics was more than three times higher in the MW sites than in the LE sites and more than two times higher than in the HE sites (Fig. 5f).

## 4 Discussion

Environmental variables greatly shape the distribution of ferns [6,14]. We found that the habitat types of *O. cinnamomeum* in Korea vary greatly on the basis of their environmental variables. RMANOVA showed that most environmental variables were significantly different depending on habitat type (Table 3). DCCA confirmed the differences in habitat types (Fig. 2, Table 2). The DCCA axes were highly correlated with relative light intensity, elevation, annual mean temperature, monthly mean precipitation, and substrate water content (Table 2). These results are consistent with those of previous studies that have shown the distribution of ferns to be driven by substrate moisture content and elevation [1,6,14].

At the low elevation sites, obligate upland plants with desiccation-tolerant characteristics were present, such as *Athyrium monomachii*, *Athyrium yokoscense*, and *Stephanandra incisa* in the herb layer; *Toxicodendron trichocarpum*, *Lindera obtusiloba*, and *Styrax obassia* in the shrub layer; and *Quercus mongolica*, *Pinus densiflora*, and *Pinus rigida* in the tree layer (Table S1). The environmental characteristics of the LE type showed high values for temperature and slope angle, and low values for elevation, substrate water content, PO_4_–P content and Ca^2+^ content. The species–environment characteristics of the LE type are coniferous–broadleaf mixed forests of secondary vegetation typically found in low-elevation mountain regions in Korea [48,49]. Plants such as *Aconitum pseudolaeve* and *Carex siderosticta*, which grow in dry forests, and plants such as *Angelica dahurica* and *Chrysosplenium pseudofauriei*, which grow in wet environments, were found in the herb layer of HE. The environmental characteristics of the HE type (i.e., low temperature, high substrate water content, and NO_3_–N content) could explain why its co-occurring species are different to those of the LE type. HE showed the characteristics of a shade-tolerant forest with *Quercus acutissima* and *Q. mongolica* in the tree and sparse shrub layers (Table S1). The community structure and environmental characteristics of HE can be described as broad-leaved forests that are in a wet and temperate environment [49]. Wet-environment indicator plants, such as *Thelypteris palustris*, *Juncus effusus*, and *Gentiana triflora*, co-occurred in MW, and the co-occurrence of shrubs and trees was sparse. The DCCA results in May showed that MWs were separated by substrate water content, substrate fertility, and precipitation (Fig. 2); however, the DCCA results in August were grouped together after the summer rainy season and showed high substrate water content, fertility, and relative light intensity (Fig. 2). The species–environment characteristics of MWs are characterized as mountain wetland vegetation rarely found in Korea [50,51]. The three habitat types showed differences in species composition and environmental variables in the DCCA ordination diagram (Fig. 2), and the results of the environmental characteristics of the three significantly differentiated habitat types in the RMANOVA support this (Table 3).

Among the environmental characteristics, light availability is one of the primary factors fueling the differentiation of the studied fern habitats [14,52]. Analysis of the community structure showed different light intensities in the *O. cinnamomeum* populations in the three habitat types (Table 3). The light availability of understory plants in forests is negatively correlated with species richness and diversity [53]. The low relative light intensity and high species richness and diversity in HE sites suggest that they provide a more competitive habitat for light availability than do LE and MW sites. In understory species, coniferous–broadleaf mixed forests exhibit a light availability greater than that of broadleaf forests [31,53], and LE has higher light availability than HE. MWs were characterized by wet meadows and wetlands on the edge of forests and had the most abundant light availability. All three types had significantly higher species richness, evenness, and diversity in August than in May (Table 3), and the height and coverage of *O. cinnamomeum* also increased (Fig. 4b and c). Competition for light availability within a plant community also increased more in summer than in spring.

Depending on the differences in community structures and environmental characteristics between habitat sites, important variables controlling population growth can vary [1,12,14], and different variables between habitat types also control the growth of *O. cinnamomeum*. Specifically, we found that *O. cinnamomeum* grew the least in LE sites (Fig. 5); this habitat was mainly characterized by high temperatures and low substrate water content. The frond area and length of the *O. cinnamomeum* population in the HE sites were the highest among the three habitat types; low temperature, relative light intensity, and high substrate water content were the main characteristics of this habitat type. Low light availability increases investment in leaf area and length [54], but leads to poor carbon fixation and reduced investment opportunities for reproduction [1]. The *O. cinnamomeum* population in HE sites showed lower biomass and fertile frond counts than did those in MW sites (Fig. 5e and f). The reproduction of dimorphic ferns has a carbon cost higher than that of other ferns [55]. *O. cinnamomeum* consumes 30 % more carbon to produce fertile fronds than sterile fronds [21], similar to the carbon costs of angiosperms [56]. The carbon produced in the frond is stored in the underground rhizomes of *O. cinnamomeum* and promotes the development of fertile fronds the following year [21]. The low number of fertile fronds in HE sites indicates that the below-ground carbon is not sufficient for reproduction (Fig. 5f). However, the high dry weight and relatively small frond area of *O. cinnamomeum* in MW sites suggests that frond thickness is developed by sufficient light (Fig. 5e). Opportunities for investment in growth and reproduction under contrasting light conditions are high in bright habitats [57], and *O. cinnamomeum* in MW have these opportunities. As a result, the growth and reproduction of *O. cinnamomeum* in Korea are controlled by temperature, moisture, and light availability in low-elevation mountains. Occasionally, *O. cinnamomeum* populations exhibiting normal growth can be found in low-elevation wetlands in North America [58], but in Korea, this plant only thrives at high elevations [17,33]. War and industrialization in the 1960s disrupted and destroyed most low-elevation wetlands in South Korea [16]. Therefore, historically, *O. cinnamomeum* may have thrived in moist lowlands in the past, and our results could be a consequence of wetland destruction at lower elevations. Currently, the source population of *O. cinnamomeum* in Korea is restricted to high-elevation montane wetlands. The dispersion of spores in ferns stems from source populations in optimal habitats [10]. In disturbed and dry low-elevation montane forests, *O. cinnamomeum* exists as a sink population dependent on spore influx. In light-poor, high-elevation montane forests, dispersal can be achieved primarily through rhizome-derived clonal growth [59].

The height of ferns inhabiting wetlands is a good indicator of their competitive ability [60]. Understanding the height of *O. cinnamomeum* and its associated species can facilitate insights into their competitiveness. The mean height *O. cinnamomeum* was, on average, 51, 78, and 87 cm in LE, HE, and MW sites, respectively, in August, the peak growing season (Fig. 4c). Low-elevation wetlands in Korea are dominated by emergent macrophytes such as *Phragmites australis, Zizania latifolia*, and *Typha angustifolia*, with an average height of 150–200 cm [61]. Emergent macrophytes with high heights reduce light availability for co-occurring species [62], thus weakening the development potential of *O. cinnamomeum* in low-elevation wetlands. However, small vascular plants, such as *Persicaria thunbergia*, *Thelypteris palustris,* and *Juncus effusus* occur frequently in high-elevation montane wetlands in Korea [63], and their average heights in our study were 23, 33, and 66 cm, respectively. The height of *O. cinnamomeum* in the montane wetlands was competitive, with sufficient light availability (Fig. 4c). A literature review found that the *O. cinnamomeum* communities reported in montane wetlands support this hypothesis (Table S4). *O. cinnamomeum* could become a dominant community in montane wetlands, depending on the wetland development [23]. The distribution area of *O. cinnamomeum* in the Sowhangbyungsan-neup wetland of Odaesan National Park increased from 15 % to 50 % over 14 years, and from less than 1 %–15 % in the Jogaedong-neup wetland [64]. Dominant species play an important role in species composition and environmental changes [65], and their roles have not been considered in the montane wetlands of *O. cinnamomeum*. In addition, the mean substrate water content in the habitat of *O. cinnamomeum* was 39 % in low-elevation mountain forests and 49 % in high-elevation mountain forests (Table S2), whereas the mean substrate water content in forests with similar vegetation ranged from 22 % to 29 % [11]. *O. cinnamomeum* communities occur in wet environments as pioneers [22], and can be used as indicators of a wet microenvironment. Monitoring wetland ferns can help predict wetland dehydration and environmental changes [13]. Over the past 100 years, the average temperature of Korea has increased by over 1.7 °C [66]. Rising temperatures and anthropogenic disturbances accelerate the dehydration of montane wetlands, reducing wetland areas and carbon storage [67]. The germination ability of the gametophytes of *O. cinnamomeum* has been shown to be reduced under high temperatures [68]; in this context of ongoing climate trends, the populations of this fern in montane wetlands will likely decrease sharply. Heavy rains and droughts triggered by changes in precipitation frequency due to climate change weaken the survival rate of fern gametophytes [14]. This decrease in habitat availability causes habitat fragmentation and limits dispersal [69]. The study of small populations of ferns can play an important role in conservation and understanding species’ ecological needs [7].

## 5 Conclusions

At the regional scale, the occurrence of *O. cinnamomeum* is driven by elevation and environmental characteristics. In Korea, important factors distinguishing the habitat types of *O. cinnamomeum* are relative light intensity, elevation, temperature, precipitation, and substrate water content. DCCA confirmed that habitats of the fern of interest can be classified as follows: low-elevation mountains, high-elevation mountains, and montane wetlands. The growth and reproductive characteristics of *O. cinnamomeum* varied across the elucidated three habitat types. The low substrate water content and high temperatures in low-elevation mountains are expected to inhibit the establishment and growth of *O. cinnamomeum*. In high-elevation mountains, low temperatures, anthropogenic disturbances, and high substrate water content promote their growth, but poor light availability limits their development to source populations. However, we conclude that montane wetlands are their optimal habitat, based on evidence of increased investment opportunities in reproduction of *O. cinnamomeum* achieved through sufficient light availability, and significant gains in the number of fertile fronds. Therefore, the growth and reproduction characteristics of *O. cinnamomeum*, across different habitat types in Korea, are driven by light availability and substrate water content. In addition, montane wetlands are the optimal habitat for *O. cinnamomeum*, as they catalyze its optimal development and population growth. However, montane wetlands are threatened by climate change and anthropogenic disturbances. This means the conservation of ferns is intimately tied to the preservation of this habitat type. This study provides habitat-linked information that can be channeled toward the conservation of *O. cinnamomeum* populations in Korea.

## Funding statement

This research was supported by the Basic Science Research Program through the 10.13039/501100003725National Research Foundation of Korea (NRF) funded by the 10.13039/501100002701Ministry of Education (NRF–2021R1I1A2041895) and by 10.13039/501100003654Korea Environment Industry & Technology Institute (KEITI) through ‘Wetland Ecosystem Value Evaluation and Carbon Absorption Value Promotion Technology Development Project’, funded by Korea 10.13039/501100003562Ministry of Environment (MOE) (RS-2022-KE002025).

## Data availability statement

Data will be made available on request.

## CRediT authorship contribution statement

**Ho Yeong Yu:** Writing – original draft, Methodology, Investigation, Formal analysis, Data curation, Conceptualization. **Jae Geun Kim:** Writing – review & editing, Validation, Supervision, Methodology, Funding acquisition, Conceptualization.

## Declaration of competing interest

The authors declare the following financial interests/personal relationships which may be considered as potential competing interests: Jae Geun Kim reports financial support was provided by Korea 10.13039/501100002701Ministry of Education. Jae Geun Kim reports financial support was provided by Korea 10.13039/501100002701Ministry of Environment. If there are other authors, they declare that they have no known competing financial interests or personal relationships that could have appeared to influence the work reported in this paper.
