# Supplementary material for: Osmundastrum cinnamomeum population differences in three habitat types in South Korea
Source: Heliyon. 2024 Jun 16;10(12):e33183. doi: 10.1016/j.heliyon.2024.e33183 (PMC11253051; doi:10.1016/j.heliyon.2024.e33183)
Supplement: Multimedia component 1 [file mmc1.docx]

***Osmundastrum cinnamomeum* population differences in three habitat types in South Korea**

**Ho Yeong Yu^1^, Jae Geun Kim^1,2*^**

^1^Department of Biology Education, Seoul National University, Seoul, 08826, Republic of Korea

^2^Center for Education Research, Seoul National University, Seoul, 08826, Republic of Korea

**Supplementary data**

**Table S1.** Detrended canonical correspondence analysis species list. Frequency of habitat soil wetness was based on the findings of Choung et al. (2021).

| Number | Layer | species | Frequency | Habitat type | |
| --- | --- | --- | --- | --- | --- |
|  |  |  |  | May | August |
| 1 | Shrub | Toxicodendron trichocarpum (Miq.) Kuntze | OBU | LA1, LA2 | LA1, LA2 |
| 2 | Herb | Stephanandra incisa (Thunb.) Zabel var. incisa | OBU | LA2, LA4 | LA2, LA4 |
| 3 | Herb | Clerodendrum trichotomum Thunb. | OBU |  | LA1, LA3 |
| 4 | Tree | Pinus rigida Mill. | OBU | LA1 | LA1 |
| 5 | Herb | Anemone narcissiflora L. | OBU |  | LA3 |
| 6 | Tree | Castanea crenata Siebold & Zucc. | OBU | LA2 | LA2 |
| 7 | Shrub | Prunus sargentii Rehder | OBU | LA1 | LA1 |
| 8 | Herb | Zanthoxylum schinifolium Siebold & Zucc. | OBU | LA1 | LA1 |
| 9 | Shrub | Lindera obtusiloba Blume | OBU | LA1, LA3, LA4 | LA1, LA3, LA4 |
| 10* | Herb | Lindera obtusiloba Blume | OBU | LA1, LA4 | LA1, LA4 |
| 11 | Tree | Pinus densiflora Siebold & Zucc. | OBU | LA3, LA4 | LA3, LA4 |
| 12 | Herb | Osmunda claytoniana L. | FACW | LA1 | LA1 |
| 13 | Herb | Callicarpa japonica Thunb. | OBU | LA4 | LA4 |
| 14 | Herb | Lespedeza maximowiczii C.K.Schneid. | OBU | LA4 | LA4 |
| 15 | Tree | Quercus serrata Thunb. ex Murray | OBU | LA4 | LA4 |
| 16 | Herb | Oplismenus undulatifolius (Ard.) Roem. & Schult. | FAC | LA1, LA2, LA3, LA4 | LA1, LA2, LA3, LA4 |
| 17 | Shrub | Styrax obassia Siebold & Zucc. | OBU | LA1, LA3, LA4 | LA3, LA4 |
| 18* | Herb | Styrax obassia Siebold & Zucc. | OBU | LA3, LA4 | LA3, LA4 |
| 19 | Herb | Athyrium monomachii Kom. | OBU | LA1, LA2 | LA1, LA2, LA3 |
| 20 | Herb | Smilax sieboldii Miq. | OBU | LA1, LA4 | LA1, LA4 |
| 21 | Herb | Dryopteris chinensis (Baker) Koidz. | OBU | LA1, LA3, LA4, HE1 | LE1, LE3, LE4, HE1, HE2 |
| 22 | Shrub | Symplocos chinensis for. pilosa (Nakai) Ohwi | OBU | LE2, HE1 | LE2, HE1 |
| 23 | Herb | Ainsliaea acerifolia Sch.Bip. | OBU | LE3, HE2 | LE3, HE2, MW2 |
| 24 | Herb | Polygonatum odoratum var. pluriflorum (Miq.) Ohwi | OBU | LE4, HE2 | LE1, LE4, HE2 |
| 25 | Tree | Fraxinus mandshurica Rupr. | FAC | LE2, LE4, HE1, MW1 | LE2, LE4, HE1, MW1 |
| 26** | Shrub | Fraxinus mandshurica Rupr. | FAC | LE2, HE1, MW1 | LE2, HE1, MW1 |
| 27* | Herb | Fraxinus mandshurica Rupr. | FAC | LE2, HE1 | LE2, LE4, HE1 |
| 28 | Herb | Athyrium yokoscense (Franch. & Sav.) H.Christ | OBU | LE1, LE2, LE3, LE4, HE2, MW3 | LE1, LE2, LE3, LE4, HE2, MW1, MW3 |
| 29 | Herb | DucheLA2ea indica (Andr.) Focke | FAC | LE1, HE2 | LE1 |
| 30 | Herb | Rubus crataegifolius Bunge | OBU | LE1, LE4 | LE1, LE4, HE2, HE3 |
| 31 | Herb | Carex curta Gooden. | OBW | LE4, HE3, MW2 | LE2, LE4, HE3, MW2 |
| 32 | Herb | Disporum smilacinum A.Gray | OBU | LE3, HE2, HE3, MW2 | LE1, LE2, LE3, HE2, HE3, MW2 |
| 33 | Shrub | Rhododendron mucronulatum Turcz. | OBU | LE1, LE2, HE1 | LE1, LE2, HE1 |
| 34* | Herb | Rhododendron mucronulatum Turcz. | OBU | LE1, HE1 | LE1, HE1 |
| 35 | Herb | Molinia japonica Hack. | FACW | LE1, MW3 | LE1, MW3 |
| 36 | Tree | Quercus mongolica Fisch. ex Ledeb. | OBU | LE1, LE2, HE1, HE3, MW1, MW2 | LE1, LE2, HE1, HE3, MW1, MW2 |
| 37 | Herb | Quercus mongolica Fisch. ex Ledeb. | OBU | LE3, LE4, HE2, MW2 | LE1, LE3, LE4, HE2, MW2 |
| 38 | Herb | Pseudostellaria heterophylla (Miq.) Pax ex Pax & Hoffm. | OBU | LE3, HE3 | HE3 |
| 39 | Herb | Viola tokubuchiana var. takedana (Makino) F. Maek. | OBU | LE3, HE2 | LE3, HE2 |
| 40 | Herb | Isodon excisus (Maxim.) Kudo | OBU | LE4, HE2, HE3 | LE4, HE2, HE3 |
| 41 | Herb | Viola acuminata Ledeb. | FACU | LE4, HE3 | LE4, HE3 |
| 42 | Tree | Quercus acutissima Carruth. | OBU | LE1, HE1, HE2 | LE1, HE1, HE2 |
| 43 | Herb | Artemisia stolonifera (Maxim.) Kom. for. stolonifera | OBU | LE3, HE3 | HE3 |
| 44 | Herb | Hypericum ascyron L. | FAC | HE3 | HE3 |
| 45 | Herb | Saussurea grandifolia Maxim. | OBU | HE1 | HE1, HE3 |
| 46 | Herb | Chrysosplenium pseudofauriei H. Lév. | FACW | HE1 | HE1, HE3 |
| 47 | Herb | Hosta capitata (Koidz.) Nakai | FAC | HE3 | HE3 |
| 48 | Herb | Angelica purpuraefolia Chung | FACU | HE3 |  |
| 49 | Herb | Aconitum pseudolaeve Nakai | OBU | HE3 | HE1, HE3 |
| 50 | Herb | Angelica dahurica (Fisch. ex Hoffm.) Benth. & Hook.f. ex Franch. & Sav. | FACW | HE1, HE3 | HE1, HE3 |
| 51 | Herb | Actaea dahurica (Turcz. ex Fisch. & C.A.Mey.) Franch. | OBU | HE3 | HE3 |
| 52 | Herb | Asarum sieboldii Miq. | OBU | LE1, LE3, HE1, HE3, MW2 | HE3 |
| 53 | Herb | Actinidia arguta (Siebold & Zucc.) Planch. ex Miq. var. arguta | FACU |  | HE3 |
| 54 | Herb | Athyrium niponicum (Mett.) Hance | OBU | HE1, HE3 | LE1, HE1, HE3, MW1 |
| 55 | Herb | Ostericum sieboldii (Miq.) Nakai, 1942. | FAC |  | HE3 |
| 56 | Herb | Aconitum jaluense Kom. subsp. jaluense | OBU | HE3, MW3 | HE2, HE3, MW3 |
| 57 | Herb | Pedicularis resupinata for. albiflora (Nakai) W.T.Lee | OBU | HE3, MW2 | HE3, MW2 |
| 58 | Herb | Micranthes octopetala (Nakai) Y.I. Kim & Y.D. Kim | OBU | HE1 | HE1, HE2, MW2 |
| 59 | Herb | Astilbe rubra Hook.f. & Thomson var. rubra | OBU | LE1, HE1, HE3, MW2, MW3 | LE1, HE1 |
| 60 | Herb | Carex siderosticta Hance | OBU | LE4, HE1, HE2, HE3, MW1, MW2, MW3 | LE3, LE4, HE1, HE2, HE3, MW1, MW2 |
| 61 | Herb | Angelica decursiva (Miq.) Franch. & Sav. | FAC | LE1, HE1, MW3 | LE1, HE1, HE3, MW3 |
| 62 | Herb | Veratrum oxysepalum Turcz. | FAC | HE1, HE3, MW1, MW2, MW3 | HE1 |
| 63 | Herb | Persicaria thunbergii (Siebold & Zucc.) H.Gross ex Nakai | OBW | MW3 | LE1, MW2, MW3 |
| 64 | Herb | Ligularia fischeri (Ledeb.) Turcz. | FAC | MW2 | LE4, MW2 |
| 65 | Herb | Angelica polymorpha Maxim. | FACW | MW3 | HE1, MW3 |
| 66 | Shrub | Acer pseudosieboldianum (Pax) Kom. | OBU | HE1, HE2, MW1, MW2 | HE1, HE2, MW1, MW2 |
| 67* | Herb | Acer pseudosieboldianum (Pax) Kom. | OBU | LE3, LE4, HE2, MW2, MW3 | LE3, LE4, HE2, MW2 |
| 68 | Herb | Caltha palustris L. var. palustris | OBW | HE1, MW3 | HE1, MW3 |
| 69 | Herb | Maianthemum bifolium (L.) F.W.Schmidt | OBU | HE3, MW2 | HE1, HE3, MW2 |
| 70 | Herb | Impatiens textori var. textori | FACW |  | HE1, MW3 |
| 71 | Tree | Maackia amurensis Rupr. & Maxim. var. amurensis | OBU | HE1, MW1 | MW1 |
| 72 | Herb | Equisetum arvense L. | FAC | LE1, MW1, MW2, MW3 | LE1, LE4 |
| 73 | Herb | Thelypteris palustris (Salisb.) Schott | FACW | LE1, HE3, MW1, MW2 | HE3, MW1, MW2, MW3 |
| 74 | Herb | Ostericum maximowiczii (F.Schmidt) Kitag. ex Maxim. | FAC | MW2, MW3 | MW3 |
| 75 | Herb | Ostericum praeteritum Kitag. | FACW | MW2 | MW2, MW3 |
| 76 | Shrub | Salix gracilistyla Miq. | FACW | MW2, MW3 | MW2, MW3 |
| 77 | Herb | Juncus effusus var. decipiens Buchenau | OBW | MW1 | MW1, MW2, MW3 |
| 78 | Herb | Gentiana triflora var. japonica (KuLA2.) H.Hara | FAC | MW1, MW2 | MW1, MW2, MW3 |
| 79 | Herb | Sedum kamtschaticum Fisch. & Mey. | OBU | MW3 |  |
| 80 | Herb | Spiraea salicifolia L. | JGW | MW1 | MW1 |
| 81 | Herb | Persicaria muricata (MeiLA2.) Nemoto | FACW |  | MW1, MW3 |
| 82 | Herb | Persicaria sagittata (L.) H.Gross ex Nakai | FACW | MW2 | MW1, MW3 |
| 83 | Herb | Solidago virgaurea subsp. asiatica Kitam. ex H. Hara | OBU | MW2 | MW2, MW3 |
| 84 | Herb | Carex onoei Franch. & Sav. | FACW | MW1 | MW1 |
| 85 | Herb | Scirpus wichurae var. asiaticus (Beetle) T.Koyama | OBW |  | MW1, MW3 |
| 86 | Herb | Carex heterolepis Bunge | OBW | MW1, MW2 | MW1, MW2 |
| 87 | Herb | Carex sabynensis Less. ex Kunth | OBU | MW3 | MW3 |
| 88 | Herb | Eleocharis wichurae Boeck. | OBW | MW1 | MW1 |
| 89 | Herb | Spiraea fritschiana Schneid | OBU | MW1, MW3 | MW3 |
| 90 | Herb | Bistorta incana (Nakai) Nakai ex T. Mori | OBU | MW3 | MW3 |

**Table S2.** One-way ANOVA results for environmental properties of habitat types. Values are shown as mean ± standard error.

| Environment variables | | Habitat types | | | F |
| --- | --- | --- | --- | --- | --- |
|  |  | LE (n=26) | HE (n=13) | MW (n=17) |  |
|  | Annual mean temperature (℃) | 10.69 ± 0.17^c^ | 8.55 ± 0.06^b^ | 7.31 ± 0.11^a^ | 139.85*** |
|  | Monthly mean precipitation (mm) | 98.35 ± 1.18^a^ | 101.51 ± 0.27^a^ | 112.77 ± 3.16^b^ | 16.09*** |
|  | Altitude (m) | 240.00 ± 29.19^a^ | 1082.54 ± 64.77^b^ | 1016.06 ± 39.89^b^ | 146.21*** |
|  | Slope angle (°) | 26.81 ± 1.49^b^ | 13.54 ± 2.77^a^ | 8.65 ± 2.53^a^ | 22.69*** |
| May | Relative light intensity (%) | 6.75 ± 2.95^a^ | 4.69 ± 1.35^a^ | 49.47 ± 9.55^b^ | 19.27*** |
|  | Water content (wt%) | 39.13 ± 1.46^a^ | 48.40 ± 2.81^a^ | 67.68 ± 5.06^b^ | 23.29*** |
|  | Organic content (%) | 16.37 ± 1.54^a^ | 19.05 ± 1.79^a^ | 40.01 ± 7.44^b^ | 9.58*** |
|  | pH | 4.19 ± 0.03^a^ | 4.61 ± 0.08^b^ | 4.47 ± 0.06^b^ | 19.00*** |
|  | Electric conductivity (µS cm^-1^) | 61.60 ± 3.47^b^ | 67.87 ± 12.83^b^ | 29.06 ± 1.52^a^ | 11.28*** |
|  | NH_4_–N (mg kg^-1^) | 20.84 ± 3.22 | 60.06 ± 31.83 | 37.59 ± 7.01 | 2.01 |
|  | NO_3_-N (mg kg^-1^) | 0.43 ± 0.12^a^ | 2.26 ± 0.76^b^ | 1.52 ± 0.65^ab^ | 3.93* |
|  | PO_4_-P (mg kg^-1^) | 3.76 ± 0.27^a^ | 8.19 ± 1.00^b^ | 10.06 ± 1.62^b^ | 12.95*** |
|  | Ca^+^ (mg kg^-1^) | 316.97 ± 57.47^a^ | 1300.98 ± 232.34^b^ | 1292.88 ± 296.96^b^ | 10.25*** |
|  | K^+^ (mg kg^-1^) | 223.13 ± 8.9^a^ | 497.92 ± 94.47^b^ | 595.16 ± 108.95^b^ | 8.99*** |
|  | Na^+^ (mg kg^-1^) | 15.55 ± 2.29^a^ | 24.02 ± 4.69^ab^ | 51.98 ± 13.60^b^ | 6.49** |
|  | Mg^+^ (mg kg^-1^) | 87.64 ± 10.97^a^ | 354.78 ± 72.57^b^ | 292.29 ± 74.34^b^ | 8.57** |
| August | Relative light intensity (%) | 6.16 ± 1.71^a^ | 3.17 ± 0.54^a^ | 43.72 ± 9.67^b^ | 17.13*** |
|  | Water content (wt%) | 28.88 ± 1.60^a^ | 43.12 ± 2.36^b^ | 71.82 ± 4.55^c^ | 61.64*** |
|  | Organic content (%) | 15.6 ± 1.26^a^ | 18.85 ± 1.28^a^ | 43.97 ± 6.90^b^ | 16.57*** |
|  | pH | 4.29 ± 0.03^a^ | 4.52 ± 0.03^b^ | 4.54 ± 0.03^b^ | 21.68*** |
|  | Electric conductivity (µS cm^-1^) | 48.08 ± 2.27^c^ | 33.75 ± 2.35^b^ | 22 ± 1.10^a^ | 41.87*** |
|  | NH_4_–N (mg kg^-1^) | 11.36 ± 2.32^ab^ | 2.32 ± 0.60^a^ | 15.01 ± 3.66^b^ | 4.55* |
|  | NO_3_-N (mg kg^-1^) | 0.06 ± 0.04^a^ | 1.60 ± 0.73^b^ | 0.74 ± 0.41^ab^ | 4.21* |
|  | PO_4_-P (mg kg^-1^) | 3.14 ± 0.24^a^ | 4.86 ± 0.75^a^ | 14.56 ± 2.52^b^ | 20.24*** |
|  | Ca^+^ (mg kg^-1^) | 281.59 ± 51.58^a^ | 1511.15 ± 187.18^b^ | 1972.07 ± 473.6^b^ | 12.63*** |
|  | K^+^ (mg kg^-1^) | 125.00 ± 5.50^a^ | 172.22 ± 13.02^a^ | 513.34 ± 125.51^b^ | 10.18*** |
|  | Na^+^ (mg kg^-1^) | 11.67 ± 1.11^a^ | 17.45 ± 1.94^a^ | 84.41 ± 22.18^b^ | 11.69*** |
|  | Mg^+^ (mg kg^-1^) | 82.04 ± 10.83^a^ | 372.22 ± 64.99^b^ | 385.3 ± 92.05^b^ | 10.76*** |

The different letters indicate statistically different groups based on Scheffe’s post hoc test (*p* < 0.05); * *p* < 0.05, ***p* < 0.01, *** *p* < 0.001.

**
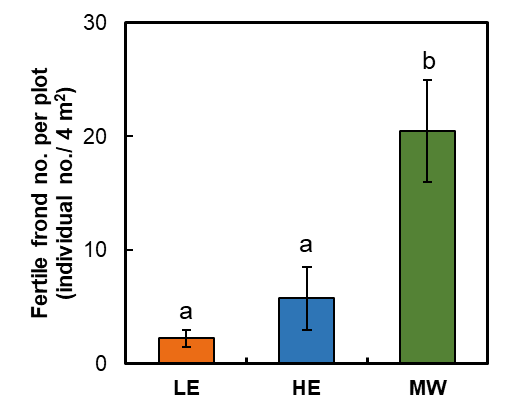
**

**Fig. S1.** Dry weight of *Osmundastrum cinnamomeum* per plot in each habitat types.

**Table S3.** Location information of the Automatic Weather Station (AWS) used in this study.

| Study site | Site elevation (m) | Site Location | AWS elevation (m) | AWS Location |
| --- | --- | --- | --- | --- |
| Incheon | 47–60 | 37°40'N 126°29'E | 48 | 37°39‘57“N 126°28‘13“E |
| Uijeongbu | 187–230 | 37°42'N 127°04'E | 100 | 37°45‘42“N 127°10‘11“E |
| Seongnam | 409–418 | 37°24'N 127°03'E | 600 | 37°26‘43“N 126°57‘50“E |
| Jecheon | 372–388 | 37°12'N 127°58'E | 822 | 37°13‘53“N 128°4‘48“E |
| Hongcheon | 971–988 | 37°50'N 128°19'E | 552 | 37°46‘41“N 128°23‘50“E |
| Inje | 675–695 | 37°43'N 128°21'E | 575 | 37°36‘44“N 128°22‘38“E |
| Jeongseon | 1288–1311 | 37°09'N 128°54'E | 822 | 37°13‘11“N 128°49‘17“E |
| Hongcheon | 772–792 | 37°50'N 128°33'E | 1019 | 37°52‘45“N 128°30‘52“E |
| Pyeongchang | 1168–1180 | 37°46'N 128°40'E | 772 | 37°40‘38“N 128°43‘6“E |
| Pyeongchang | 1049–1058 | 37°46'N 128°42'E | 772 | 37°40‘38“N 128°43‘6“E |

**Table S4.** Elevation and co-occurring species of *Osmundastrum cinnamomeum* East Asia and North America as reported in published literature.

| Location | Site | Wetland type | Elevation (m) | Co-occurring species | Reference |
| --- | --- | --- | --- | --- | --- |
| Connecticut, USA | Yale-Myers | Marsh | 170–300 | *Maianthemum canadense*, *Symplocarpus foetidus* | Green and Duguid 2020 |
| Illinois, USA | Gensburg | Marsh | 184 | *Osmunda spectabilis*, *Thelypteris palustris* | Gonzalez et al. 2019 |
| Indiana, USA | Tamarack Bog | Bog | 444 | *Carex trisperma*, *Rubus hispidus* | Bender et al. 2012 |
| North Carolina, USA | Martins Fork | Bog | 720 | *Osmunda regalis*, *Sphagnum palustre* | Thompson et al. 2012 |
| North Carolina, USA | Four Level | Bog | 750 | *Glyceria striata*, *Impatiens capensis* | Thompson et al. 2012 |
| North Carolina, USA | Kentenia | Bog | 790 | *Dichanthelium dichotomum*, *Lycopus virginicus* | Thompson et al. 2012 |
| North Carolina, USA | Tulula Bog | Bog | 800 | *Carex stricta*, *Rubus hispidus* | Warren et al. 2007 |
| North Carolina, USA | Boone Fork Bog | Bog | 1036 | *Scirpus spp.*, *Sphagnum spp.* | Pittillo 1994 |
| North Carolina, USA | Long Hope Valley bog | Bog | 1418 | *Sphagnum spp.* | Pittillo 1994 |
| New York, USA | Bewkes | Swamp | 410 | *Osmunda regalis* | Britton and Watkins 2016 |
| Quebec, Canada | Hemlock Carr | Swamp | 410 | *Betula alleghaniensis*, *Onoclea sensibilis*, *Tsuga canadensis* | Flinn et al. 2008 |
| Pyeongchang, Korea | Mt. Odae wetland | Montane fen | 792–1180 | *Thelypteris palustris*, *Juncus effusus*, *Gentiana triflora* | This study |
| Mungyeong, Korea | Bongamsa wetland | Montane marsh | 500 | *Osmunda japonica* | Oh et al. 2011 |
| Uljin, Korea | Mt. Cheonchug wetland | Montane marsh | 560 | *Carex lanceolata*, *Vicia unijuga*, *Smilax nipponica* | Lim et al. 2020 |
| Nagano, Japan | Nyukasa Marsh | Montane marsh | 1737 | *Carex omiana*, *Scirpus wichurae* | Maki and Shimano 2021 |

**Reference**

Choung, Y. et al. Categorized wetland preference and life forms of the vascular plants in the Korean Peninsula. J. Environ. Ecol. 45, 1-6. https://doi.org/10.1186/s41610-021-00183-0 (2021).

Green, L. & Duguid, M. Vascular plant diversity of forested wetlands in southern New England. *Rhodora* **122**, 73-101. https://doi.org/10.3119/19-21 (2020).

Pittillo, J. D. Vegetation of three high elevation southern Appalachian bogs and implications of their vegetational history. *Water Air Soil Pollut.* **77**, 333-348 (1994).

Flinn, K. M., Lechowicz, M. J. & Waterway, M. J. Plant species diversity and composition of wetlands within an upland forest. *Am. J. Bot.* **95**, 1216-1224. https://doi.org/10.3732/ajb.0800098 (2008).

Oh, H.-K., Han, Y.-H. & Choi, S.-H. Conservation management methods and classification type of flora in the Bongamsa (Temple) area, Mungyeong. *Korean J. Environ. Ecol.* **25**, 447-469 (2011).

Maki, R. & Shimano, K. Species composition and environmental factors of wetland vegeta-tion in Nyukasa Marsh, Nagano Prefecture, Japan. *Japan J. Veg. Sci.* **38**, 1-16 (2021).
